# Evaluation of the Propensity score methods for estimating marginal odds ratios in case of small sample size

**DOI:** 10.1186/1471-2288-12-70

**Published:** 2012-05-30

**Authors:** Romain Pirracchio, Matthieu Resche-Rigon, Sylvie Chevret

**Affiliations:** 1 Service de Biostatistique et Information Médicale, Hôpital Saint-Louis, UMR-S717 Inserm; Sorbonne Paris Cité, Université Paris Diderot, 1 avenue Claude Vellefaux, Paris 75010, France; 2 3- Service d'Anesthésie-Réanimation, Hôpital Européen Georges Pompidou, Université Paris 5 Descartes, Sorbonne Paris Cité, Paris, France

**Keywords:** Propensity scores, Propensity score matching, Inverse probability weighting, Small sample size, Simulation

## Abstract

**Background:**

Propensity score (PS) methods are increasingly used, even when sample sizes are small or treatments are seldom used. However, the relative performance of the two mainly recommended PS methods, namely PS-matching or inverse probability of treatment weighting (IPTW), have not been studied in the context of small sample sizes.

**Methods:**

We conducted a series of Monte Carlo simulations to evaluate the influence of sample size, prevalence of treatment exposure, and strength of the association between the variables and the outcome and/or the treatment exposure, on the performance of these two methods.

**Results:**

Decreasing the sample size from 1,000 to 40 subjects did not substantially alter the Type I error rate, and led to relative biases below 10%. The IPTW method performed better than the PS-matching down to 60 subjects. When N was set at 40, the PS matching estimators were either similarly or even less biased than the IPTW estimators. Including variables unrelated to the exposure but related to the outcome in the PS model decreased the bias and the variance as compared to models omitting such variables. Excluding the true confounder from the PS model resulted, whatever the method used, in a significantly biased estimation of treatment effect. These results were illustrated in a real dataset.

**Conclusion:**

Even in case of small study samples or low prevalence of treatment, PS-matching and IPTW can yield correct estimations of treatment effect unless the true confounders and the variables related only to the outcome are not included in the PS model.

## Background

In non-randomized studies, any estimated association between treatment and outcome can be biased because of the imbalance in baseline covariates that may affect the outcome. In this context, propensity score methods (PS) [1] are increasingly used to estimate marginal causal treatment effect. The propensity score, as defined by Rosenbaum and Rubin [1] is the individual probability of receiving the treatment of interest conditional on the observed baseline covariates. It has been demonstrated that, within the strata of subjects matched on the propensity score, distributions of these covariates tend to be similar between treated and untreated [1]. Therefore, conditioning on the propensity score allows to draw unbiased marginal estimates of treatment effects [1].

Four methods of using the propensity score have been so far described: stratification [1,2], adjustment [1,2], matching [1-4] and more recently inverse probability of treatment weighting (IPTW) [3,5-9]. Using an empirical case study and Monte Carlo simulations, several authors [8,10] recently showed that the PS-matching and the IPTW more efficiently reduced the imbalance in baseline covariates than the two other methods did. However, these methods were evaluated using large simulated datasets of about 10,000 observations, and roughly balanced treatment groups [10]. From a practical point of view, if propensity scores have usually been applied to large observational cohorts [11-13], they have been also used in the setting of small samples [14,15] or with important imbalances in the treatment allocation, as observed, for instance, when estimating the benefit of intensive care unit (ICU) admission [16].

Although PS-matching and IPTW have not been evaluated in the context of small sample sizes, such a situation raises specific questions. First, because the propensity score is used to balance baseline covariates more than to predict treatment assignment, it has been recommended [17-19] to include in the PS model all the potential confounders and to avoid any selection procedure based on the goodness-of-fit [19,20]. However, the limited sample size restricts the number of variables to be included in the PS regression model to limit model *over parameterization*. Moreover, in case of small sample sizes, it is not clear whether one PS method, i.e. PS matching or IPTW, outperforms the other or not, considering, on one hand, that matching without replacement might lead to a further decrease in the sample size (and thus, in the statistical power of outcome comparisons), and, on the other hand, that IPTW might give an excessive weight to some observations that could dramatically influence the results. All these points could be similarly addressed in case of important imbalances in the size of the treatment arms. Therefore, our goal was to explore such specific situations in order to provide some warnings concerning the use of PS methods, for analysts, but also for readers.

Actually, we assessed the performance of PS-matching and IPTW in the particular context of small samples, when the odds ratio (OR) is used as the measure of treatment effect. We present the results of Monte Carlo simulations in which we studied the influence of the sample size, the prevalence of treated patients, and the strength of the association between the variables and the outcome and/or the treatment exposure, in order the assess the accuracy of PS methods in terms of bias, variance estimation and Type I error rates in the estimation of treatment effect. Finally, some illustration is provided from a real observational dataset, assessing the benefit of allogeneic stem cell transplantation in a small sample of patients with multiple myeloma.

## Methods

### Monte carlo simulation study

Monte Carlo simulations were used to evaluate the performance of PS-matching and IPTW to estimate the marginal OR of treatment effect in the context of small sample sizes and/ or low prevalence of the treated population. They consisted in (1) randomly generated N independent datasets in several settings defined by sample size, treatment effect and covariates effect on both treatment and outcome; (2) applying the PS-matching and IPTW approaches to analyze the data, separately. In each setting, the performance of each approach was assessed by computing the bias, the mean squared error (MSE) and the variance of the estimated OR from the N replications of the dataset. Type I and Type II error rates, as defined by the frequency of rejecting the null or alternative hypothesis under the null or the alternative, respectively, were also estimated.

#### Data-generating process

Let Z be the variable defining treatment allocation (Z = 1 for the treated, 0 otherwise), Y be the outcome of interest (Y = 1 for those subject who experienced the outcome, 0 otherwise) and X a set of 4 independent and identically distributed baseline covariates $\left(X_{j},j=1,\ldots ,4\right)$.

The probability of allocated treatment and that of experiencing the outcome were described by the two following logistic models, respectively:

(1) $$\text{log}it(P(Z_{i}=1{\text{|X}}_{\text{i}}))=\alpha_{0}+\alpha_{1}X_{i1}+\ldots +\alpha_{4}X_{i4}$$

(2) $$\text{log}it(P(Y_{i}=1|Z_{i},X_{i}))=\beta_{0}+\beta_{T}Z_{i}+\beta_{1}X_{i}{}_{1}+\ldots +\beta_{4}X_{i4}$$

where $Z_{i}$ was the treatment assignment for subject *i*, $\left(\alpha_{0},\ldots ,\alpha_{4}\right)$ and $\left(\beta_{0},\beta_{T},\ldots ,\beta_{4}\right)$ the sets of corresponding slope and regression coefficients. Regression coefficients allowed considering different situations of covariate association with the treatment and the outcome: $X_{1}$ did not affect any of them (α_1_ = β_1_ = 0), $X_{2}$was associated only with the outcome (α_2_ = 0, β_2_ = b), $X_{3}$ only with the treatment (α_3_ = a, β_3_ = 0), and $X_{4}$ with both as a true confounder (α_4_ = a, β_4_ = b).

The value of the intercept $\alpha_{0}$ was successively set at 0 and −1.38 to guarantee a marginal probability of treatment allocation of 0.50 and 0.2, respectively. For each set of values of ( $\beta_{T}$, a, b), the value of $\beta_{0}$ was determined by a minimization algorithm from a sample of 1,000,000 in order to guarantee the outcome to occur in 50 per cent of subjects, approximately.

For each subject, treatment allocation and outcome were randomly generated from Bernoulli distributions with subject-specific probability of treatment assignment derived from equation (1) or equation (2), as the success probability, respectively. Each covariate was randomly generated from a Normal distribution N(μ = 0, σ = 0.5).

Several situations were examined, that differed in terms of:

– Sample size, ranging from 1,000 down to 40 (1000, 900, 800, 700, 600, 500, 400, 300, 200, 180, 160, 140, 120, 100, 80, 60, 40)

– Treatment effect $\beta_{T}$, successively fixed at 0 (null hypothesis), 0.41 and 0.92 (alternative hypotheses of moderate or strong treatment effect) corresponding to conditional ORs fixed at 1, 1.5 and 2.5, respectively

– Strength of the association between the covariates and both the treatment and the outcome, with a and b fixed at 0.41 and 0.92, corresponding to moderate and strong association, respectively.

### Analysis of simulated data sets

#### Propensity score models

The propensity score models the probability that a given patient would be exposed to the experimental treatment, conditionally to his(her) baseline covariates [1]:

(3) $$\text{log}it(P(Z=1|V))=\hat{\beta }V$$

where $\hat{\beta }$ is the maximum likelihood estimator of the baseline covariate effects, and *V* is the vector of covariates included in the model. Eight models were examined according to the vector *V*: 3 univariable models with either one of the covariates *X*_*2*_*, X*_*3*_*, X*_*4*_ (models 1, 2, 3, respectively), 3 bivariate models (*X*_*2,*_*X*_*3*_), (*X*_*2,*_*X*_*4*_) and (*X*_*3,*_*X*_*4*_) (models 4, 5, 6, respectively), one 3-covariate model with *X*_*2*_*, X*_*3*_*, X*_*4*_ (model 7) and finally the full model with *X*_*1*_*, X*_*2*_*, X*_*3*_*, X*_*4*_ (model 8).

The propensity score (*PS*_*i*_) of the patient *i* was then estimated from the predicted probability of treatment given his(her) covariates as obtained by logistic regression. The PS-matching method was used because it has been proposed as a reference method when using propensity score [10]. However, because it has been demonstrated that this approach may not be strictly unbiased to estimate a marginal OR [21], we also applied the IPTW approach which has been shown to be unbiased for estimating marginal ORs [22].

#### Propensity score based matching

Different types of matching algorithms have been proposed [23,24]. We used 1–1 matching without replacement. Each treated subject was randomly selected and then matched to the nearest untreated subject based on calipers of width of 0.2 of the standard deviation of the logit of the PS, as previously recommended [23,24].

#### Inverse-probability-of-treatment weighting

Each subject was weighted in the likelihood using the inverse estimated probability of treatment actually administered, *z*_*i*_, as follows [6]:

(4) $$IPTW_{i}=\frac{z_{i}}{PS_{i}}+\frac{1-z_{i}}{1-PS_{i}}$$

Note that, for treated subjects (*z*_*i*_ = 1), $IPTW_{i}=\frac{1}{PS_{i}}$, while for untreated (*z*_*i*_ = 0), $\;IPTW_{i}={\frac{1}{1-PS}}_{i}$.

### Treatment effect estimates

In each simulated dataset, the benefit of treatment on the outcome was first estimated by fitting a logistic model applied to the PS-matched dataset using generalized estimating equations with robust variance estimator (package gee for R, Vincent J Carey, Thomas Lumley and Brian Ripley). Then, a weighted logistic model using a generalized linear model adapted to data from a complex survey design, with inverse-probability weighting and design-based standard errors applied (package svyGLM for R, Thomas Lumley).

### Model performance criteria

A total of 7,300 independent datasets – generated as described above – was required to detect a difference in type I error of at least 0.005 as compared to 0.05 with a power of 95%. The performance of each of the 8 PS models was evaluated from those 7,300 simulated sets using the following criteria: type I error, statistical power, absolute and relative biases from the marginal OR (%) and mean square error (MSE). Type I error and statistical power were estimated by the proportions of true and false null hypotheses that were rejected, respectively. MSE was computed by the average of the squares of the differences between the estimate and the true value fixed by simulation.

All simulations and statistical analyses were performed using R software version 1.10.1 ( http://www.R-project.org) running on a Linux platform.

## Results

### Simulation results

#### Full fitted models

To evaluate the impact of small sample sizes on estimation, we first fitted a non-parsimonious PS model, including all the four baseline covariates (model 8).

When using the PS-matching approach, the mean number of pairs ranged from 21.2 to 22.6 (i.e., from 53.0 to 56.5% of the sample) for 40 patients and increased up to 370.0 - 421.2 (74.0-84.2%) for 500 patients.

**Figure 1:**
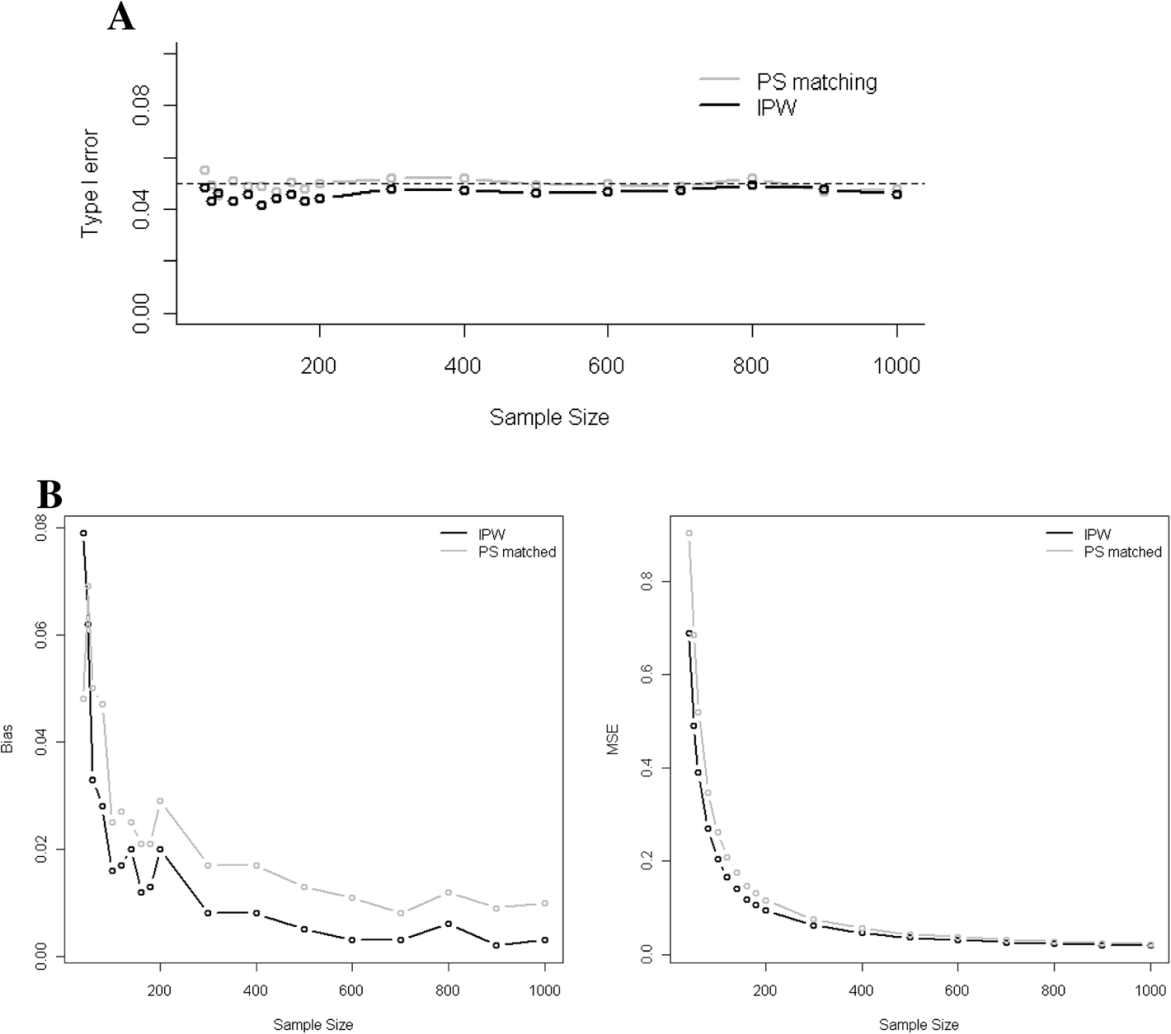
**Evolutions of the type I error (Panel A) and the bias and the mean square error (MSE) in the estimated coefficients (Panel B) when decreasing the sample size according to the method used, analysis of PS matched or inversely probability weighted (IPW) data sets.** These results were obtained using a non parsimonious PS model that included the four baseline covariates. The strength of the association between the baseline covariates, the treatment and the outcome was uniformly set as strong (odds ratio of 2.5) with a marginal prevalence of the treatment of 0.5. In the upper panel (Panel A), the type I error rate was obtained under the null hypothesis. In the lower panel (Panel B), the bias and the mean square error were computed using a treatment effect set at 2.5.

Under the null hypothesis, no substantial increase in the Type I error rate was observed as the sample size decreased from 1,000 down to 40 subjects. As shown in Figure 1 (Panel A), for a treatment effect and a strength of the association between the covariates and the outcome both set at a strong level, and a marginal prevalence of the treatment at 0.5, the Type I error rate ranged from 0.039 to 0.052 for PS matching, and from 0.036 to 0.047 for IPTW. The Type I error rate was not markedly affected by the strength of the association between the covariates and the treatment/outcome (Table 1), nor by treatment prevalence, decreasing from 0.5 down to 0.2 (data not shown).

**Table 1:** Type I errors, Bias and Mean Square Error (MSE) for non parsimonious PS model according to the method used (PS matching (PSm) or IPTW) and to the strength of the association between baseline covariates, treatment/outcome

**N**		**OR(a) = 1.5, OR(b) = 1.5**		**OR(a) = 2.5, OR(b) =1.5**		**OR(a) = 1.5, OR(b) = 2.5**		**OR(a) = 2.5, OR(b) = 2.5**	
		**PSm**	**IPTW**	**PSm**	**IPTW**	**PSm**	**IPTW**	**PSm**	**IPTW**
**40**	**Type I error**	0.055	0.048	0.053	0.056	0.052	0.039	0.052	0.047
	**Bias**	0.05	0.057	0.049	0.07	0.059	0.055	0.048	0.079
	**%**	5.6	6.5	5.4	7.8	7.1	6.6	5.7	9.4
	**Variance**	0.875	0.593	0.918	0.694	0.873	0.576	0.903	0.683
	**MSE**	0.878	0.597	0.92	0.699	0.876	0.579	0.905	0.690
**60**	**Type I error**	0.045	0.046	0.047	0.046	0.044	0.039	0.044	0.039
	**Bias**	0.058	0.036	0.058	0.049	0.048	0.022	0.05	0.033
	**%**	6.4	4	6.4	5.4	5.7	2.6	6	3.9
	**Variance**	0.511	0.36	0.553	0.414	0.484	0.329	0.516	0.388
	**MSE**	0.514	0.362	0.556	0.416	0.486	0.33	0.519	0.389
**100**	**Type I error**	0.049	0.046	0.047	0.046	0.047	0.034	0.04	0.036
	**Bias**	0.023	0.018	0.02	0.022	0.021	0.015	0.025	0.016
	**%**	2.6	2	2.2	2.5	2.5	1.8	2.9	1.9
	**Variance**	0.254	0.194	0.282	0.223	0.242	0.177	0.261	0.204
	**MSE**	0.255	0.194	0.283	0.223	0.243	0.177	0.262	0.204
**500**	**Type I error**	0.049	0.046	0.05	0.048	0.041	0.038	0.042	0.037
	**Bias**	0.01	0.007	0.012	0.008	0.01	0.005	0.013	0.005
	**%**	1.1	0.8	1.3	0.9	1.2	0.6	1.5	0.6
	**Variance**	0.04	0.034	0.045	0.038	0.037	0.032	0.042	0.035
	**MSE**	0.04	0.034	0.045	0.038	0.037	0.032	0.042	0.035

This table specially details the sample sizes ranging from 40 to 500. Prevalence of the treatment was set at 0.5. Conditional treatment effect was set at log(2.5) except for Type I errors estimations (log(1)). (N: number of subjects; PS: propensity score; IPTW: inverse probability of treatment weighting, OR(a) and OR(b): strengths of the association between baseline covariates and treatment or outcome respectively, as defined on an odds ratio scale).

Given a strong treatment effect and balanced treatment groups (treatment prevalence set at 0.5), the bias and the mean square error expectedly increased, as long as the sample size decreased, for both PS-matching and IPTW (Table 1 & Figure 1, Panel B). However, even for sample sizes of less than 100 subjects, bias remained below 10% (Table 1). For sample sizes of more than 60 subjects, IPTW estimations were systematically less biased and MSE smaller than those reached by PS-matching. Similar results were found in case of low prevalence of the treatment.

When the strength of the association between the covariates and the treatment/outcome decreased, IPTW estimators remained similarly or even less biased than PS-matching estimators, down to 60 subjects (Table 1). However, when the sample size was set at 40, the PS-matching estimators outperformed better than the IPTW estimators.

Whatever the method, the reduction of treatment effect (with OR decreasing from 2.5 to 1.5) was associated with a global decrease in the bias and the MSE, but with similar relative bias (data not shown). In this situation, the largest bias was observed for both methods when the number of subjects decreased to less than 60 (IPTW: relative bias: 8.1%, MSE: 0.675; PS-matching: relative bias: 7.6%, MSE: 0.933).

As expected, the variance in the estimation of treatment effect increased monotonically while the sample size decreased. The variances of the IPTW estimators were systematically smaller than the variances of the PS estimators. For both methods and whatever the treatment effect, the smallest variance was observed when baseline covariates were strongly associated to the outcome, but moderately to treatment.

#### Selected fitted models

When the simulations were fitted using PS-models that included at least *X*_*4*_, no substantial increase in the Type I error rate was observed as the sample size decreased from 1,000 to 40 subjects whatever the strength of the association between the covariates, the treatment and the outcome. However, when removing the true confounder from the PS model, the Type I error rate substantially increased to a maximum obtained for the IPTW method, in case of strong association between the two remaining covariates, the treatment and the outcome (Table 2). Moreover, the IPTW method seems always more conservative than the PS-matching.

**Table 2:** Bias and variance of the estimated treatment effect for the different selected PS models and according to the method used (PS matching or IPTW)

		**Model 1**		**Model 2**		**Model 3**		**Model 4**		**Model 5**		**Model 6**		**Model 7**		**Model 8**	
**N**		**X2**		**X3**		**X4**		**X2,X3**		**X2,X4**		**X3,X4**		**X2,X3,X4**		**X1,X2,X3,X4**	
		**PSm**	**IPTW**	**PSm**	**IPTW**	**PSm**	**IPTW**	**PSm**	**IPTW**	**PSm**	**IPTW**	**PSm**	**IPTW**	**PSm**	**IPTW**	**PSm**	**IPTW**
**40**	**Type I error**	0.062	0.041	0.059	0.051	0.058	0.040	0.060	0.047	0.053	0.036	0.048	0.045	0.053	0.044	0.052	0.047
	**Bias**	0.273	0.235	0.265	0.251	0.086	0.06	0.259	0.25	0.075	0.061	0.064	0.071	0.075	0.074	0.048	0.079
	**Relative bias**	32.6	28.1	31.7	30	10.3	7.2	31	29.9	9	7.3	7.6	8.5	9	8.8	5.7	9.4
	**Variance**	0.809	0.508	0.872	0.566	0.862	0.551	0.874	0.573	0.843	0.56	0.907	0.634	0.904	0.644	0.903	0.683
	**MSE**	0.884	0.563	0.942	0.629	0.869	0.555	0.941	0.635	0.848	0.563	0.911	0.639	0.909	0.649	0.905	0.69
**60**	**Type I error**	0.055	0.050	0.058	0.057	0.045	0.038	0.052	0.053	0.043	0.037	0.049	0.042	0.044	0.041	0.044	0.039
	**Bias**	0.239	0.203	0.235	0.217	0.041	0.02	0.245	0.219	0.047	0.022	0.051	0.028	0.059	0.03	0.05	0.033
	**Relative bias**	28.6	24.3	28.1	25.9	4.9	2.4	29.3	26.2	5.6	2.6	6.1	3.3	7.1	3.6	6	3.9
	**Variance**	0.466	0.317	0.52	0.354	0.494	0.339	0.532	0.348	0.477	0.333	0.524	0.379	0.514	0.375	0.516	0.388
	**MSE**	0.523	0.359	0.575	0.401	0.495	0.339	0.592	0.396	0.479	0.334	0.527	0.38	0.517	0.376	0.519	0.389
**100**	**Type I error**	0.062	0.057	0.065	0.066	0.052	0.039	0.057	0.062	0.042	0.034	0.046	0.041	0.044	0.037	0.04	0.036
	**Bias**	0.209	0.193	0.212	0.203	0.029	0.013	0.215	0.203	0.029	0.013	0.028	0.016	0.028	0.016	0.025	0.016
	**Relative bias**	25	23.1	25.3	24.3	3.5	1.6	25.7	24.3	3.5	1.6	3.3	1.9	3.3	1.9	2.9	1.9
	**Variance**	0.239	0.175	0.254	0.194	0.242	0.186	0.256	0.188	0.239	0.18	0.259	0.205	0.253	0.199	0.261	0.204
	**MSE**	0.282	0.212	0.299	0.235	0.243	0.186	0.302	0.23	0.24	0.18	0.259	0.205	0.254	0.2	0.262	0.204
**500**	**Type I error**	0.148	0.155	0.150	0.162	0.051	0.045	0.146	0.157	0.046	0.040	0.046	0.042	0.045	0.037	0.042	0.037
	**Bias**	0.187	0.182	0.192	0.191	0.014	0.005	0.193	0.191	0.015	0.005	0.014	0.005	0.014	0.005	0.013	0.005
	**Relative bias**	22.3	21.8	22.9	22.8	1.7	0.6	23.1	22.8	1.8	0.6	1.7	0.6	1.7	0.6	1.5	0.6
	**Variance**	0.037	0.032	0.042	0.036	0.04	0.034	0.041	0.034	0.039	0.033	0.044	0.037	0.042	0.035	0.042	0.035
	**MSE**	0.072	0.066	0.079	0.072	0.04	0.034	0.078	0.071	0.039	0.033	0.044	0.037	0.042	0.035	0.042	0.035

This table specially details the sample sizes ranging from 40 to 500. Prevalence of the treatment set at 0.5, treatment effect.

**Figure 2:**
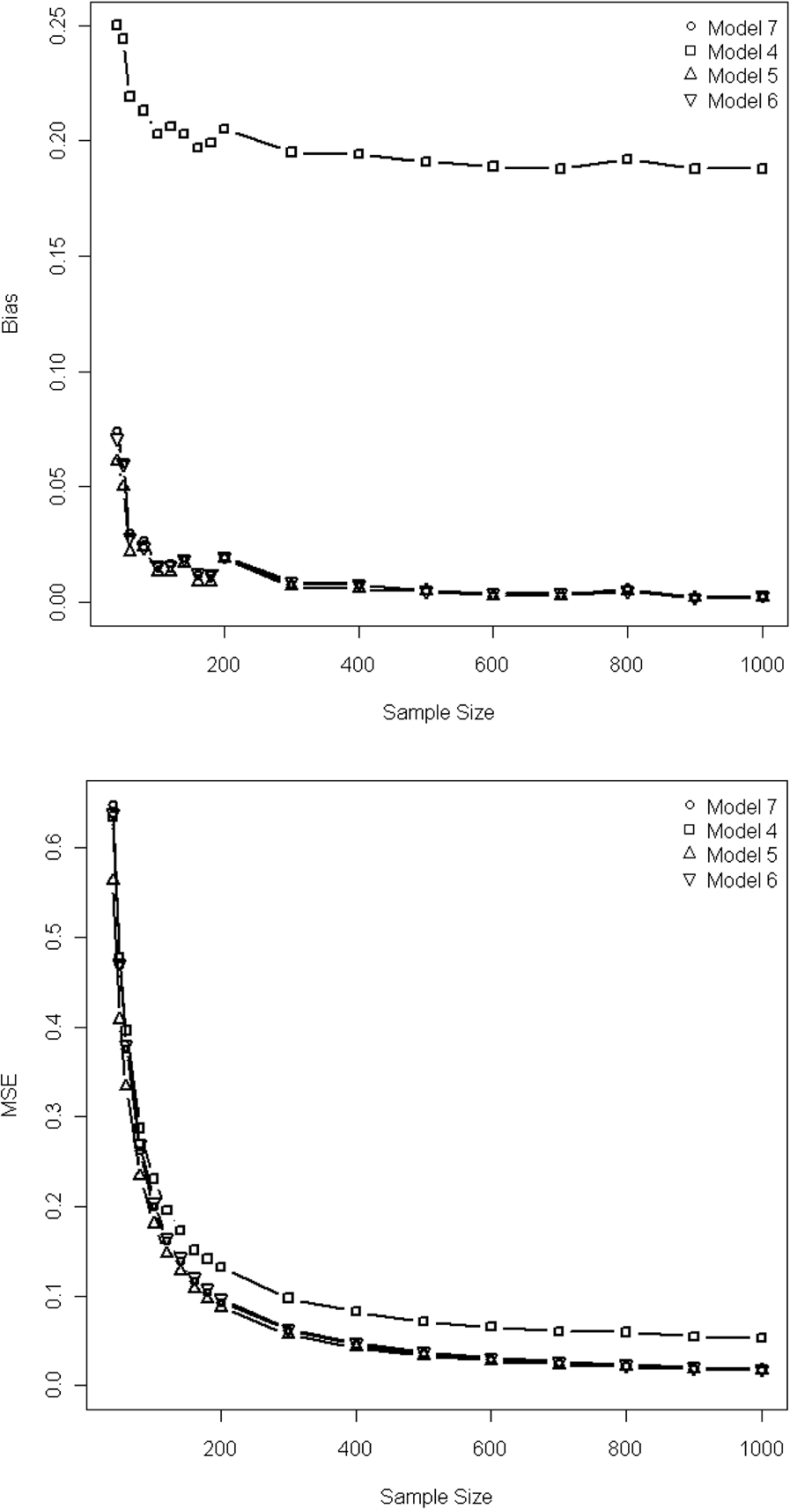
**OR biases and MSE according to the sample size and variables included in the propensity score when using an IPTW approach.** Upper panel: OR biases; Lower panel: OR Mean Square Error (MSE).

In case of strong treatment effect, variables included in the PS model affected the bias and the MSE: non-inclusion of the true confounder systematically implied a bias larger than 10%; moreover the inclusion of variables unrelated to the treatment allocation but related to the outcome allowed to achieve less biased results than that of variables related to treatment but unrelated to the outcome (Table 2 & Figure 2). Such impact on bias was more important with IPTW than with PS-matching (Table 2), and when the covariates and the treatment/outcome were strongly rather than moderately associated. The bias, variances and MSE of the IPTW estimators were systematically smaller than those of the PS estimators. Similar trends were found when the treatment effect was moderate, but the absolute values of both biases and MSE were smaller than those observed with a strong treatment effect.

The variance of the estimated treatment effect decreased when the true confounder or covariate unrelated to the treatment but related to the outcome were included in the PS, especially with the IPTW method (Table 2). Adding to model 5 (including true confounder + variable related to the outcome) a variable related to treatment allocation (model 7) did not reduce the bias, but increased the variance of the estimation. Moreover, adding to the PS model a variable related neither to the treatment nor to the outcome (model 8), did not further improve the precision of the estimation, but increased the variance, especially when the sample size decreased below 100.

Similar results than those observed using a non-parsimonious PS model, were found when the marginal prevalence of the treatment decreased from 0.5 to 0.2.

For both IPTW and PS-matching methods, the minimally biased estimation was obtained by incorporating in the PS model, as well as true confounders, variables strongly associated to the outcome but moderately associated to the treatment.

### Illustration to a real observational dataset

To illustrate these results, we then applied the PS methods described above in a real situation, where we aimed at evaluating the benefit of sequential autologous-allogeneic tandem approach in Multiple Myeloma (MM), using a small observational dataset [15]. Twenty-three patients (median age 48 years, range 26–59 years) with relapsed multiple myeloma (MM) who received the treatment under study were compared to a control group of 142 MM relapsing patients free of such a treatment (median age 51.5 years, range 25–65 years). Hence, this dataset combined the two situations of relatively small sample size (n = 165) and very low prevalence of treatment (23/165, 14%). We used the survival status at 24 months as the outcome measure, with benefit of treatment measured on ORs.

Three baseline variables, related to treatment allocation, to the outcome or to both of them, were available at baseline: 1) age at diagnosis, only associated with treatment allocation (with untreated patients more likely to be older than treated; p = 0.05), 2) beta2 microglobulin level ≥ 3.5, only moderately associated with the outcome OR = 1.8 (95%CI 0.9;3.5 , p = 0.10), and 3) time elapsed since the first line treatment at relapse, strongly associated with both the treatment allocation OR = 0.4 (95%CI 0.2;0.8 , p = 0.01) and to the outcome OR = 0.4 (95%CI 0.2;0.7 , p = 0.002). Of note, log time was considered instead of the time to insure the validity of logistic models.

In this situation, we applied the PS-matched and PS-weighted (IPTW) approaches, when PS incorporated the baseline information separately. Results are summarized in Table 3. As expected by the simulation results, the choice of the variable included in the PS model heavily impacted the estimation of treatment effect, and this was even more pronounced when using the IPTW-weighted estimator than the PS-matched estimator. Indeed, consistently with the simulation findings, including only the variable related to the treatment, e.g. *age at diagnosis*, yielded estimates different than that obtained by including only the true confounder, namely *time elapsed since the first line treatment at relapse*.

**Table 3:** Estimated odds ratios (OR) of death and 95% confidence interval using naive, propensity score matching, or IPTW approaches

**Model**	**Adjustment in the original set**	**PS-matched sample**		**IPT-Weighted sample**	
**Covariates**	**OR (95%CI) p-value**	**No pairs**	**OR (95%CI) p-value**	**Sum of weights**	**OR (95%CI) p-value**
**X1 = age**	0.44 (0.14;1.42) p = 0.17	22	0.27 (0.06;1.23) p = 0.091	327.5	0.39 (0.11;1.32) p = 0.13
**X2 = beta2micro**	0.47 (0.15;1.47) p = 0.19	23	0.27 (0.05;1.41) p = 0.12	330.0	0.48 (0.14;1.65) p = 0.25
**X3 = time to relapse**	0.24 (0.07;0.84) p = 0.026	23	0.19 (0.05;0.65) p = 0.0088	349.1	0.27 (0.08;0.92) p = 0.039
**X1 + X2**	0.49 (0.15;1.57) p = 0.23	22	0.48 (0.09;2.58) p = 0.39	317.9	0.41 (0.12;1.36) p = 0.15
**X1 + X3**	0.22 (0.06;0.85) p = 0.028	18	0.20 (0.03;1.17) p = 0.073	456.0	0.23 (0.05;1.00) p = 0.052
**X2 + X3**	0.26 (0.07;0.93) p = 0.039	23	0.19 (0.05;0.68) p = 0.011	340.8	0.26 (0.08;0.86) p = 0.028
**X1 + X2 + X3**	0.24 (0.06;0.93) p = 0.040	20	0.41 (0.08;2.1) p = 0.28	432.1	0.21 (0.05;0.86) p = 0.031

If we assumed from the former simulation results that the model including the variable related to the outcome and the true confounder (namely, the *beta2 microglobulin level* and the *time elapsed since the first line treatment at relapse*) was the optimal model, results were concordant with those obtained by simulation. Hence, the results obtained by including only the true confounder were very close to the former in terms of estimates and variance. Omitting from the PS model either the true confounder or the variable related to the outcome substantially modified the estimation of treatment effect.

Moreover, as demonstrated in the simulation study, adding to the PS model variables only related to the treatment allocation (that is, models with age, beta2 microglobulin level and time to relapse) led to a larger variance in the estimation of the treatment effect, especially when using a PS-matching approach.

Finally, if considering the recommended strategy defined above, that is, only using covariates strongly associated with the outcome in the PS, the conclusion was somewhat concordant whatever the approach.

## Discussion

Propensity score methods have been widely studied to analyze very large datasets [11-13]. However, although originally developed in epidemiological settings with large sample sizes, they are increasingly being used to estimate causal treatment effects in clinical settings, where sample sizes are rather limited as compared to the former settings. Actually, while the former epidemiological settings deal with thousands of patients, the clinical setting usually have to deal with at most several hundred patients, or even less than 50 [14,15]. However, only a few publications have addressed the issue of PS properties in such a situation [25]. Thus, the aim of this study was to get further insights into their performances when either the sample sizes or the prevalence of exposure are rather low, and to address the question of the variables to include in the PS model in such situations.

To answer those questions, we used the 2 main recommended PS approaches [11-13], namely the PS-matching and IPTW. The large use of PS matching was confirmed by a PubMed search (performed on October, 2010) that selected 521 references dealing with PS matching from a whole set of 2,045 PS references. By contrast, only 64 references dealt with IPTW or PS-weighted approaches, and this could be explained because they have been more recently promoted, and appear more complex to use than PS-matching. Other PS approaches have been developed such as PS adjustment [1,2] or PS-quintile stratification [1,2] but they have been shown of less performance in large samples. Thus, it appeared unlikely that they would perform better in the context of small sample size or low prevalence of exposure.

Based on a simulation study, we showed that no substantial increase in the Type I error rate was observed as the sample size decreased from 1,000 to 40 subjects, but that small sample sizes lead to biased estimations of the marginal treatment effect. However, relative bias remained inferior to 10%, even with small sample down to 40 patients. Of note, in case of small sample sizes down to 60 subjects, IPTW method seems to perform always better than the PS-matching. Such results could have been expected as IPTW method preserves the sample size all along the analysis process and maximizes the available amount of information as compared to PS-matching. On the contrary, 1:1, without replacement matching procedures are associated with a reduction in the sample size, as all treated usually cannot find a non-treated to be matched with. Hence, because the weighted dataset is generally larger than the matched dataset, the variance and the confidence intervals associated with the IPTW estimations are expected to be smaller. However, when N was set at 40, the PS-matching estimators were either similarly or even less biased than the IPTW estimators. One possible explanation is that, in case of very small samples, the weighting resulted in a significant distortion of the population, with excessive weights given to marginal subjects. This was illustrated in the real dataset where the sum of weights was sometimes far above the actual sample size. However, this could be addressed by using stabilized weights, as previously reported [26]. We thus reran the analyses using such stabilized weights, but in our case, stabilization did not affect the results (data not shown).

The second question addressed in this study was the selection of the covariates to be included in the PS model, in case of small sample sizes. Previous simulation studies have addressed different questions concerning the choice of the variables to be included in the PS model, such as the effect of omitting confounding variables when using quintile-stratified propensity adjustment in longitudinal studies [27], or the relative performances of PS models when including variables related to treatment allocation, variables related to outcome or all variables related to either outcome or treatment or neither [28]. However, data concerning appropriate PS models when dealing with limited sample sizes are still lacking. Indeed, while it is usually recommended [17-19] to include in the PS model all the potential confounders, this could lead to *over parameterized* PS models when the number of treated is limited. On the other hand, it has been previously reported that PS model misspecification could highly influence the estimation [25,27]. Therefore, in the context of small sample sizes, one might consider preferable to apply some variable selection procedure, but it seems crucial to adequately choose those variables to be included in the PS model. To do so, it has previously been shown that, because the goal of a PS model is to efficiently control confounding, but not to predict treatment or exposure, the use of selection procedure based on the goodness-of-fit cannot be recommended [19,20,25]. As previously reported [17,25], we found that the inclusion in the PS model of variables unrelated to the exposure but related to the outcome is mandatory. Indeed, consistently with the results published by Brookhart et al. [25], we found that the inclusion of such variables decreased both the bias and the variance of the estimated treatment effect. We also found that excluding the true confounder from the PS model resulted, whatever the method used, in a significantly biased estimation of treatment effect. These results are not in line with the advices provided by Brookhart et al. [25]. Indeed, the latter authors suggested that including in the PS model a true confounder that is only weakly related to the outcome, but very strongly related to the exposure might result in a loss in efficiency that is not offset by a large enough decrease in bias. Our different results might be otherwise explained by the fact that our simulation study did not explore a situation where the true confounder is very strongly related to the exposure but only very weakly to the outcome.

We measured the performance of each approach using type I error, power, bias and MSE estimated from independent replicates of simulated datasets. However, in practice, the accurate way to evaluate the performance of a PS procedure relies on the assessment of its ability to reach conditional exchangeability between groups, as recommended by Austin [4,29]. Balance is commonly measured on standardized differences, though permutation tests have been also reported as an interesting alternate way of evaluation. Indeed, such tests tend reject when bias due to inexact propensity matching is enough to undermine causal inferences, and tend not to reject when that bias is small enough to be ignored, could be used instead [30].

The importance of variables to be included in the model was exemplified in our real dataset, where achieved balances and treatment effect estimates (Table 3) heavily depended on the approach (PS-match versus IPTW) and the included covariates, with estimated ORs of death ranging from 0.19 (when PS included the true confounder) up to 0.4, which was reached by both IPTW and PS-matching approach in situations where the true confounder was omitted from the model. While we chose to focus on the 2 currently recommended PS methods, PS matching and IPTW, it should be emphasized that PS adjustment and stratification on the quintiles of the PS have not been and compared to the former methods in the context of small sample sizes. Further simulation studies should be performed to compare the performances of the 4 methods in the context of small sample size.

For the PS matching, we used a classical pair matching procedure, based on a caliper defined as 0.2SD of the logit of the PS. This was chosen as the mostly used matching method in clinical settings [31], while that caliper width was recommended on the basis of simulation results [23,32]. However, several other matching procedures could have been proposed that may offer interesting advantages over the former, notably in the context of small sample sizes [33]. Otherwise,data-adaptive algorithms such as ridge matching have been also reported to perform better than “classical” pair matching [34]. Nevertheless, evaluating the matching algorithm was not the goal of this paper, though its importance deserves to be the focus of a specific simulation study. This should be the framework of further studies.

We did not consider other model misspecifications but those regarding the PS model. Indeed, in the class of IPW estimators, it is well known that the weighted estimators are consistent provided that the model for estimating the PS is correctly specified. Otherwise, to relax this restriction, so-called doubly-robust estimators have been proposed [8], that also require the specification of the outcome regression model. Misspecifications may consist in choosing the wrong link function, or selecting a linear instead of a non-linear predictor. In case of model misspecification, the mean square error of estimate has been shown to be reduced by using the matching estimator, both for small-to-moderate sample sizes [35]. Further work in this topic may be of interest.

The choice of the odds ratio as a measure of treatment effect has been debated. Indeed, the choice of the appropriate PS based estimators for conditional and marginal non-linear treatment effects has been thoroughly discussed in the recent literature [21,36-38]. Actually, the problem with OR, usually described as non-collapsibility, refers to the fact that conditional and marginal effects might differ unless the true effect is null [21,39,40]. Moreover, Austin et al. [23] have previously shown that PS-matching, as compared to PS-stratification or -adjustment, offers substantially less biased estimates of both conditional and marginal OR. The choice to control the conditional treatment effect rather than the marginal as proposed by Austin [23] was driven by our wish to achieve a probability of 0.50 of experiencing the outcome and to maximize the statistical power considering the small sample size. The resulting marginal treatment effect was thereafter estimated using a second simulation study of sample size 1,000,000 that confirmed that conditional and marginal treatment effects were in the same range. Otherwise, previous simulations studies support the use of IPTW estimators as approximately unbiased estimators of marginal odds ratios [22,38]. The choice of a binary outcome and the use of an adapted and largely applied regression model were motivated by our will to overcome a biostatistical issue that has been raised by one of our clinical question.

Finally, the choice of an event rate of 0.5 could be debated. Indeed, the prevalence of event may be far from 0.5 in clinical situations. However, we chose a prevalence of 0.5 because our goal was to assess the effects of decreasing the sample size. Then, when dealing with sample size of 40–60 patients, an even rate of 0.1-0.2 would have been associated with a very small number of events, and a high risk of model misspecification. To confirm this assumption, we reran the simulation using an event rate fixed at 0.2. As expected, decreasing the event rate down to 0.2 was associated for both methods with unacceptable increases in variance and MSE, when the sample size was ≤100 (variance ranging from 1.294 to 177.4; MSE ranging from 1.309 to 177.4).

## Conclusions

In conclusion, this simulation study revealed that, even in case of small study samples or low prevalence of treatment, both propensity score matching and inverse probability of treatment weighting can yield unbiased estimations of treatment effect. However, in such situations, a particular attention should be paid to the choice of the variables to be included in the PS model. The optimal model seems to be that including the true confounder and the variable related only to the outcome, although reporting several models as a sensitivity analysis may appear a good way of arguing for or against the robustness of the estimated effects. Future work in this area should aim at providing for the clinicians: (1) formal rules to choose the best approach between matching and weighting according to the population characteristics, (2) practical strategies to select the variables for inclusion in a propensity score model in case of small study samples or low treatment prevalence.

## Abbreviations

PS: propensity score; IPTW: inverse probability of treatment weighting; MSE: mean squared error; GMM: Generalized methods of moments; OR: odds ratio; MM: multiple myeloma; 95CI: 95% Confidence interval.

## Competing interests

The authors declare that they have no competing interests.

## Authors’ contribution

RP and MRR performed the analysis and wrote the manuscript, SC supervised the analysis and the elaboration of the manuscript. All authors read and approved the final manuscript.

## Pre-publication history

The pre-publication history for this paper can be accessed here:

http://www.biomedcentral.com/1471-2288/12/70/prepub
